# Cardiac assessment accuracy by students using palm-held ultrasound compared to physical examination by skilled cardiologists: a pilot study with a single medical student

**DOI:** 10.1186/s12947-022-00277-2

**Published:** 2022-03-25

**Authors:** Shirley Sarig, Tsafrir Or, Gassan Moady, Shaul Atar

**Affiliations:** 1 Azrieli Faculty of Medicine in the Galilee, Bar-Ilan University, Safed, Israel; 2 Department of Cardiology, Galilee Medical Center, Nahariya, Israel

**Keywords:** Palm-held ultrasound, Transthoracic echocardiography, Physical examination

## Abstract

**Background:**

Despite the inherent limitations of the traditional cardiac physical examination (PE), it has not yet been replaced by a more accurate method.

**Methods:**

We hypothesized that a single medical student, following a brief training (two academic hours) with the PHU, will better identify abnormal findings including significant valvular diseases, pericardial effusion and reduced LV function, as compared to PE performed by senior cardiologists and cardiology fellows. Transthoracic echocardiogram (TTE) served as a ‘gold standard’.

**Results:**

Seventy-seven patients underwent TTE, of them 64 had an abnormal finding. PE identified 34 patients with an abnormal finding compared to 52 identified by PHU (*p* < 0.05). Ejection fraction (EF) below 50% was found in 35 patients on TTE, compared to only 15 and 6 patients by PE and PHU, respectively (*p* < 0.05). There was no difference in valvular dysfunction diagnosis detected by PE and medical students using PHU. The overall accuracy of PHU compared to TTE was 87%, with a specificity of 94% and sensitivity of 64% (the low sensitivity was driven mainly by EF assessment), whereas the accuracy of PE was 91%, specificity 91% and sensitivity 38% (again driven by poor EF assessment).

**Conclusions:**

Cardiac evaluation using PHU by a single medical student was able to demonstrate similar accuracy as PE done by cardiac specialists or cardiology fellows. The study topic should be validated in future studies with more medical students with a very brief training of cardiac ultrasound.

## Introduction

For centuries, the physical examination (PE) has been the backbone of cardiovascular diagnosis. In the past few years, studies demonstrate that PE, with the stethoscope being in the forefront, carries significant limitations. Despite those limitations and the emergence of novel miniaturized imaging devices, the stethoscope has not yet been replaced [1].

The approach to a patient with known or suspected cardiovascular disease begins with a directed history and targeted PE, which is a time honored tradition. For the last two decades, there has been a gradual decline in PE skills, from student to faculty specialist levels [2]. This raises a great concern to both clinicians and medical educators. Today, only a minority of internal medicine and family physicians correctly recognize classic cardiac findings, and despite the popular belief, this performance does not improve with experience. Moreover, it has become more difficult to teach PE, as physicians become busier and devote less time to tutor students and residents. The declining quality of medical education (especially related to clinical reasoning and supervised training of medical skills) nowadays, especially in places where medical schools are being opened without criteria or supervision by health authorities, also account for the worsening accuracy of PE. The declining skills of PE derive not only from the busy agenda of physicians, but especially from suboptimal training. As a consequence there is an increasing reliance on noninvasive imaging devices to establish the presence and severity of cardiovascular disease [2]. Lack of confidence on PE, which results in ordering unnecessary tests, is another adverse outcome. Furthermore, cardiac abnormalities such as LV dysfunction, LV thrombus and valvular vegetations are findings that cannot be assessed using PE alone [1].

For the past 50 years, conventional echocardiography is routinely used to evaluate the structure and function of the heart. Even though the equipment has improved and became lighter, it still weighs more than a 100 kg, and it takes time to move it to the bedside [3]. Hand-carried ultrasound (HCU) is a portable imaging device, battery operated, lightweight (2–3 kg) and about the size of a laptop computer. The high image resolution, low-cost, and simplicity of the HCU may modify traditional medical practice and medical education by complementing the PE with a real-time cardiovascular imaging [4]. The portable HCU extends the physician’s diagnostic capabilities beyond the limits of the PE along with a potential for a more accurate and faster diagnosis, lower cost, while extends the physician’s need of an expert operator [5, 6].

In 2005, Kobal et al. hypothesized that medical students without clinical experience could accurately evaluate and diagnose cardiac abnormalities using HCU after short training. The study compared the accuracy of cardiovascular diagnosis by medical students using HCU to PE performed by cardiologists. Of the 239 abnormal findings identified by standard echocardiography, medical students recognized 75%, whereas cardiologists identified only 49% [7].

The V-scan (GE, USA) is a novel, smaller than HCU, pocket-sized palm-held ultrasound (PHU). It is so much smaller than the HCU that it can be carried in the physician's pocket. It enables medical professionals including cardiologists, general practitioners, OB/GYN, primary care, emergency physicians and intensivists to visualize the heart in two-dimensions and in a color Doppler imaging at the point of care.

We therefore hypothesized that a single medical student, following a brief training (two academic hours) with the PHU, will better identify abnormal findings including significant valvular diseases, pericardial effusion and reduced LV function, as compared to PE performed by senior cardiologists and cardiology fellows.

## Methods

### Study design

This was a prospective cohort study, designed to compare PHU with PE in patients admitted to the hospital and referred for routine TTE.

The study was approved by the local ethics committee and conducted according to the principles established in the Helsinki declaration (NHR 067–17). All patients signed an informed consent form. All the data is available upon request.

#### Declarations

We recruited consecutive patients, age range 34–92 years, who were admitted to the Department of Cardiology for a variety of reasons – myocardial infarction, heart failure, arrhythmias, chest pain evaluation, etc. Medical history regarding diabetes, hypertension, hyperlipidemia, presence of a pacemaker, smoking, artificial valves, family history of heart disease and body mass index (BMI) was collected for each patient.

We collected data using an entry sheet containing three main categories (Table 1). We performed TTE according to international guidelines. We used either LCX 50 or EPIQ 7 (Philips, Medical Systems, Andover, Massachusetts). The TTE studies were performed by experienced technicians and experienced by board certified echocardiography experts. We randomly studied consecutive patients on several consecutive days. A cardiologist performed a targeted PE, in an attempt to define the following: 1. Left Ventricle (LV) function; 2. Presence of pericardial effusion; and 3. Significant (moderate to severe) valvular dysfunction. We defined LV dysfunction as an LVEF ≤ 50%. Otherwise, we did not determine which specific physical findings or criteria are essential to reach a conclusion regarding any of the two other clinical questions. Following the cardiologist’s PE, a medical student (after a brief training of not more than two academic hours) performed the PHU. Both the cardiologist and the student were unaware of the TTE results or the medical history of the patient (double blinded). We did not study patients in critical conditions or on ventilators. Exposure of the examiners to prior information before the test excluded the patient from the study. The primary outcome of the study was the overall accuracy of abnormal diagnoses by PE and PHU compared to TTE.

**Table 1:** Entry sheet containing three main categories for data collection

Study number:	Date:	Subject name:		
Normal cardiac examination	Yes	No		
Ejection fraction	Above 50%	Less than 50%		
Mitral regurgitation	None	Mild	Moderate	Severe
Mitral stenosis	None	Mild	Moderate	Severe
Aortic stenosis	None	Mild	Moderate	Severe
Aortic regurgitation	None	Mild	Moderate	Severe
Pericardial effusion	Yes	No		

The values were represented as means ± SE. Different lowercase letters indicate significant differences among the different sand burial depths of *X. spinosum* by using one-way ANOVA from LSD tests (*P* < 0.05)

### PHU training

We trained only one student to be the main operator, and another student to assist in interpretation. The training consisted of a 30-min introduction to cardiac ultrasound and to normal anatomy and function by TTE. We then did an additional 30 min session and went over common cardiac pathologies and LV function assessment. We then performed a 30 min bedside session to demonstrate the PHU examination protocol by an experienced operator, with an emphasis on scanning technique to obtain the cardiac views and the evaluation criteria for each pathology. Then we did a 30 min bedside instructed session in which the students performed the PHU study by themselves, to familiarize with the device and to practice obtaining all images correctly. These students were already trained in PE, yet they did not perform previous PE on the patients.

### Sample size

We enrolled 77 consecutive patients who were admitted to the cardiology department. Due to the nature of the research that focuses on measuring parameters in several ways, we identified possible measurement bias. Ultrasound is operator dependent [8]. Both the stethoscope and the PHU do not sometimes give accurate results for the tested parameters. If both testes missed a diagnosis compared with TTE as ‘gold standard’, we analyzed their diagnosis as ‘wrong’ even if both parties reached the same diagnosis. Both tests (PHU and TTE) tend to miss a minimal amount of pericardial effusion.

### Statistical analysis

Quantitative data is described with Means and standard deviation, medians and ranges. Qualitative data is presented using frequencies and percentages, 95% Confidence interval for proportions was calculated. The accuracy of qualitative data as compared to the TTE outcomes are presented by measures as Sensitivity, Specificity, Accuracy, positive predictive value (PPV) and negative predictive value (NPV). For comparing the results of both the PE and PHU with the echocardiogram results (quality variables), we used McNemar test. P value ≤ 0.05 is considered as a significant value.

## Results

### Patient population

We recruited 77 stable patients (72.7% men), age 34 to 92 years (mean age 63.7 ± 12.9), average BMI – 30.8 ± 1.8 kg/m^2^, who were admitted to the cardiology department for various etiologies and underwent TTE. Their baseline characteristics: Diabetes mellitus (38%), systemic hypertension (62%), hyperlipidemia (59%), current smoking (42%), family history of ischemic heart disease (38%).

### Echocardiographic findings

Of the 77 patients, 64 patients (83.1%) had an abnormal finding on TTE. The physical examination performed by a physician identified only 34 patients (44.2%) with an abnormal finding, with an accuracy of 55.9%. The PHU examination performed by medical students identified correctly 52 patients (67.5%), with an accuracy of 68.8%. Thus the accuracy of PHU performed by medical students was significantly higher (*p* < 0.05). The TTE findings are presented in Table 2.

**Table 2:** Echocardiographic findings

Echocardiogram results		*N* (*n* = 77) (%)	**(%)**
	Normal examination	13	(16.9)
	Abnormal examination	64	(83.1)
**Ejection fraction (%)**	≥ 50%	42	(54.5)
	< 50%	35	(45.5)
**Mitral regurgitation**	None + Mild	69	(89.6)
	Moderate + Severe	8	(10.4)
**Mitral stenosis**	None + Mild	76	(98.7)
	Moderate + Severe	1	(1.3)
**Aortic regurgitation**	None + Mild	76	(98.7)
	Moderate + Severe	1	(1.3)
**Aortic stenosis**	None + Mild	72	(93.5)
	Moderate + Severe	5	(6.5)
**Pericardial effusion**	Yes	6	(7.8)
	No	71	(92.2)

### Comparison of PE and PHU

As seen in Table 3, the ejection fraction is better identified by the PHU. An EF value ≤ 50% was found in 42.9% of the patients by the PHU, whereas the PE revealed only 17.1% of the cases. Murmurs were identified with high resemblance to TTE findings, while the physical examination resembled the TTE results with higher likelihood. Yet, the difference is small, and is probably explained by the small sample size, especially regarding aortic valve murmurs.

**Table 3:** Correct diagnoses by PHU and PE with TTE as a reference

	**TTE**	**PHU**	**PE**	**% Absolute difference (95% CI)**	***P*** **value**
Ejection fraction (%) > 50%	42	38/42 (90.5%)	35/42 (83.3%)	3%) 1%-13%)	0.508
Ejection fraction (%) < 50%	35	15/35 (42.9%)	6/35 (17.1%)	9%25%-3%))	0.035
Mitral regurgitation absent	69	64/69 (92.8%)	67/69 (97.1%)	| % -3| (1%-10%)	0.453
Mitral regurgitation present	8	4/8 (50%)	4/8 (50%)	0%	1.00
Mitral stenosis absent	76	73/76 (96.1%)	76/76 (100%)	| % -3| (1%-10%)	**+**
Mitral stenosis present	1	1/1 (100%)	0/1 (0%)	100%	**+ +**
Aortic regurgitation absent	76	70/76 (92.1%)	76/76 (100%)	-6%|| (2%-14%)	**+**
Aortic regurgitation present	1	0/1 (0%)	1/1 (100%)	-100%||	+ +
Aortic stenosis absent	72	68/72 (94.4%)	70/72 (97.2%)	| % -3.2| (1%-10.2%)	0.625
Aortic stenosis present	5	0/5 (0%)	2/5 (40%)	|-40%| (11.8%-76.9%)	+ +
Pericardial effusion absent	71	70/71 (90.9%)	71/71 (100%)	-1.4%|| (0.2%-4.0%)	**+**
Pericardial effusion present	6	0/6 (0%)	0/6 (0%)	0%	+

Absent means none or mild valvular dysfunction, while present refers to moderate to severe. The sensitivity is defined as the presence of a heart condition, while specificity is mentioned as the absence of one

*PE* physical examination, *Per. Eff.* pericardial effusion

+ Cannot be calculated

### PPV and NPV

As presented in Table 4, the NPV is almost identical in all parameters tested comparing the PE and the PHU. The PPV is slightly higher in favor of the PE regarding mitral regurgitation and aortic stenosis, but higher with the PHU with respect to mitral stenosis and pericardial effusion.

**Table 4:** PPV and NPV of the PHU and physical examination compared with echocardiographic findings

	**PHU**		**PE**	
	**PPV**	**NPV**	**PPV**	**NPV**
**EF (%)**	79%	65%	78%	64%
**Mitral regurgitation**	44%	94%	67%	94%
**Mitral stenosis**	25%	100%	-	99%
**Aortic regurgitation**	0%	99%	-	99%
**Aortic stenosis**	0%	93%	50%	96%
**Pericardial effusion**	0%	92%	-	7.7%

### Accuracy

As presented in Table 5, the accuracy is slightly higher by the PE, in all parameters except for the ejection fraction. Yet, the accuracy in both the PE and the PHU is near 90% in almost all parameters measured. The overall accuracy of PHU compared to TTE was 87%, with a specificity of 94% and sensitivity of 64% (the low sensitivity was driven mainly by EF evaluation), whereas the accuracy of PE was 91%, specificity 91% and sensitivity 38% (again driven by poor EF assessment).

**Table 5:** Accuracy of PHU and physical exmination

	**PHU vs TTE (95% CI)**	**PE vs TTE (95% CI)**
Ejection fraction (%)*	68.8% (57.8–78.1%)	67.2% (55.0–75.5%)
Mitral regurgitation	88.3% (79.0–93.9%)	92.2% (83.7–96.7%)
Mitral stenosis	96.1% (88.7–99.1%)	98.7% (92.3–100%)
Aortic regurgitation	90.9% (82.1–95.8%)	98.7% (92.3–100%)
Aortic stenosis	88.3% (79.0–93.9%)	93.5% (85.3–97.5%)
Pericardial effusion	90.9% (82.1–95.8%)	92.9% (83.7–96.7%)

^*^ *p* < 0.05 for ejection fraction ≥ 50% versus < 50%; *CI*  Confidence interval

## Discussion

Our study demonstrates that a very short training period (2 h) allowed a medical student with no prior echocardiographic skills to perform basic cardiac evaluation using PHU and achieve results almost identical to a physical examination performed by a cardiology fellow or a senior physician using a stethoscope.

The use of ultraportable echocardiography devices has been an object of study in various clinical settings of cardiology and other medical specialties. Several studies have shown an incremental benefit when hand-handled ultrasonography compliments the routine general PE [9, 8, 3].

Moreover, these days, telemedicine is a rapidly evolving field in the medical arena. PHU can allow physicians from intern to senior level, both within the hospital and in the community clinic to consult a specialist while transmitting the test data in real-time or later by saving the information on the PHU. This will allow avoiding unnecessary follow-up tests due to lack of confidence or inexperience of the attending physician and will allow for a quicker and more accurate diagnosis.

Since the findings by PHU are very much similar to the ‘gold standard’ in this study, and since the study population has very high likelihood of showing abnormalities with PHU, the results shown in our study may in a way be very much obvious. However, we did not intend to demonstrate that PHU may be used as a screening tool to detect significant valvular disease, cardiomyopathy, or pericardial disease in daily clinical practice. In this case, the study population should be general population or patients who may present with shortness of breath. We mainly intended to prove its ability to detect cardiac abnormalities even by medical students after a brief training session.

Kobal et al. [7] utilized 18 h of training medical students, yet in their study they used the Optigo hand-carried device, which is a much larger device with a wider range of imaging capabilities. Moreover, the students who performed the studies were first-year students, with minimal knowledge in cardiac anatomy and physiology. Our students were 4^th^ year students, and we used a PHU with much more limited imaging capacbilities and much easier to use. Therefore we were able to reach sufficient skills with such a short training session. In addition, Stokke et al. [10] utilized only a 4 h training session in their study, using a PHU again, and reached similar results to ours.

As demonstrated in our study and previous studies, both the PHU and physical examination will probably not be able to replace standard echocardiography as a ‘gold standard’ in the very near future. However, it can be used as an excellent tool for rapid diagnosis of cardiac function, including LV function assessment, valvular disorders and pericardial fluid with high sensitivity and specificity.

Currently, students acquire comprehensive training on how to perform a physical examination that includes the use of a stethoscope as a tool for evaluating heart sounds and cardiac function. Our study and previous studies [4, 7, 5] point to the difficulty of skilled physicians these days to diagnose cardiac abnormalities by physical examination only. Thus we strongly recommend that the use of a PHU be integrated during the professional training phase of medical students in parallel with the study of the complete and comprehensive physical examination. Moreover, with future technical improvements (the addition of Doppler measurements, improved resolution), satellite communication, and the required short training period—PHUs can become a useful and inexpensive device for providing better standard of care in low-income and remote regions of the world.

### Study limitations

This is a small size study. Nevertheless, the results obtained in our study demonstrate that not much experience or abundant knowledge is required to operate the palm-held device, and the outcomes are almost identical to those obtained by a physical examination carried out by a cardiology fellow or a senior cardiologist. The study hypothesis was indeed not proven as we expected. Yet, we did show that a single medical student, after 2 h of training session, reached similar results with the PHU as PE done by senior cardiologists. It is therefore reasonable to expect that with more experience gained by using the PHU and with overcoming the learning curve, the accuracy of PHU operated by medical students would improve.

The definition of EF by PE is not an easy task. However, this is an important daily clinical dilemma, as patients are often admitted to the ER with signs and symptoms of heart failure (HF). The clinician then needs to define whether he is treating HF with preserved or reduced EF. In our study we used similar criteria as used already by Kobal et al. [7].

It is likely that a larger sample size would have allowed for additional cardiac pathologies to be detected by the palm-held device, and emphasize its advantages. Another limitation is that the PHU is not ideal in patients with poor echocardiographic window due to obesity or chronic lung disease. PHU is currently also unable to quantify correctly the severity of valvular pathology – the current available devices do not offer Doppler measurements.

## Conclusions

The results of our study are hypothesis generating. The study topic should be validated in future studies with more medical students with a very brief training of cardiac ultrasound.

## Data Availability

The dataset supporting the conclusions of this article is available as an Excel file upon request from the corresponding author.
